# Reference Genes for Expression Analyses by qRT-PCR in *Propsilocerus akamusi* (Diptera: Chironomidae)

**DOI:** 10.3390/biology14091158

**Published:** 2025-09-01

**Authors:** Wenbin Liu, Yaning Tang, Ziming Shao, Jiaxin Nie, Xue Bai, Zhe Nie, Chunmian Liu, Yajin Zhang, Chuncai Yan, Yiwen Wang

**Affiliations:** 1 College of Life Sciences, Tianjin Normal University, Tianjin 300387, China; skylwb@tjnu.edu.cn (W.L.);; 2 Inner Mongolia Autonomous Region Agricultural and Animal Husbandry Technology Extension Center, Hohhot 010010, China; nmgbaixue@163.com; 3 School of Pharmaceutical Science and Technology, Tianjin University, Tianjin 300072, China; 4 Shanxi Key Laboratory of Nucleic Acid Biopesticides, Shanxi University, Taiyuan 237016, China

**Keywords:** *Propsilocerus akamusi*, reference genes, developmental stages, different parts, temperature treatments

## Abstract

*Propsilocerus akamusi* (Tokunaga, 1938) larvae serve as key bioindicators for water quality assessment. This study identifies optimal reference genes for RT-qPCR under diverse experimental conditions. Fifteen candidate genes commonly employed in other insect species were selected, candidate genes commonly used in other insect species. Homologous genes were identified in the *P. akamusi* genome through sequence alignment with their *Drosophila melanogaster* counterparts. Expression stability across developmental stages, body parts, temperature variations, and deltamethrin/nickel chloride exposures was systematically evaluated using geNorm, NormFinder, BestKeeper, and ΔCt methods. *RPL32* exhibited the highest expression stability across different body parts of adults under varying temperature conditions, and *RPS11* and *RPL8* showed the greatest stability across developmental stages and in larvae exposed to different temperatures. Furthermore, under nickel chloride and deltamethrin treatments, *RPS11* and *RPL8* maintained the highest expression stability. The results indicated that the expression stability of reference genes varied under different conditions. Among different body parts of adults, *RPL32* and *RPL4* exhibited the most stable expression. Across different developmental stages, *RPS11* and *RPL8* performed best in terms of expression stability. Under different temperature treatments, *RPL32* and *RPL4* remained stable in adults, while *RPS11* and *RPL8* showed the greatest stability in larvae. Similarly, under nickel chloride and deltamethrin treatments, *RPS11* and *RPL8* demonstrated the most stable expression in larvae. Through the above research, we can advance ecosystem-impact insights and bolster environmental protection and water quality monitoring.

## Simple Summary

*Propsilocerus akamusi* (Tokunaga, 1938), a key indicator species in aquatic ecosystems, plays a crucial role in assessing water pollution through gene expression studies. This study aims to identify optimal internal reference genes for quantitative real-time PCR (RT-qPCR) analysis in this species under varying developmental body stages, parts, and stress conditions, including temperature fluctuations, pesticide exposure (deltamethrin), and heavy metal exposure (nickel chloride). By evaluating the expression stability of 15 candidate genes across multiple conditions, *RPL32* and *RPL4* demonstrated the highest stability in different conditions. In contrast, *RPS11* and *RPL8* exhibited the greatest stability in larvae across developmental stages, temperature variations, and exposures to nickel and deltamethrin. These findings establish a standardized foundation for utilizing RT-qPCR technology to investigate the molecular regulatory mechanisms of *P. akamusi* under adverse conditions. This work enhances the understanding of environmental pollution impacts on ecosystems and offers scientific support for environmental protection efforts and water quality monitoring.

## 1. Introduction

*Propsilocerus akamusi* (Tokunaga, 1938) belongs to the order Diptera, family Chironomidae, called a non-biting midge [1]. Its larvae inhabit the bottom of freshwater wetlands, marshes, lakes, and slowly flowing rivers, where they play an important role in the benthic biotic community [2,3]. The larvae of *P. akamusi* also have significant economic value. They are used as feed for ornamental fish, bait for fishing, and also serve as a high-quality animal protein source for aquaculture [4,5]. In addition, *P. akamusi* larvae are sensitive to water pollutants, making them suitable as bioindicator species for water quality monitoring. This species inhabits damp environments such as freshwater wetlands and also exhibits strong tolerance to certain pollutants, such as cadmium ions, rendering it an excellent model for studying the adaptive mechanisms of organisms to pollutants [6].

As an important aquatic bioindicator species, the toxicological study of *P. akamusi* is crucial for understanding the impact of environmental pollutants on ecosystems [7]. However, previous research on *P. akamusi* has largely remained at the ecological and behavioral levels, failing to delve into the molecular level. This is mainly due to the lack of a well-established genomic research foundation for this species.

Recently, we have successfully completed the whole-genome sequencing of *P. akamusi* and achieved high-quality genome assembly at the chromosomal level. This accomplishment provides a solid data foundation for subsequent functional gene studies [8,9]. Concurrently, we have conducted annotations of the *P. akamusi* genome, with a specific focus on gene families associated with heavy metal stress response and detoxification metabolism, such as cuticular protein (CP), ATP-binding cassette (ABC) transporter genes, Glutathione S-Transferase (GST), and Cytochrome P450 (CYP) [3,4,10,11]. Through transcriptome analysis, we have also preliminarily obtained gene expression profiles of *P. akamusi* under various environmental stresses and identified a series of differentially expressed genes potentially involved in its adaptation and detoxification processes to pollutants [12].

However, the validation of transcriptome data and further investigations into gene functions, such as gene knockout and knockdown experiments, require accurate quantification of gene expression. Quantitative real-time reverse transcription polymerase chain reaction (RT-qPCR), a highly sensitive and specific technique for gene expression quantification, plays a pivotal role in this process [13]. As the method of choice for precise measurement of transcript levels, RT-qPCR provides researchers with a reliable tool for investigating gene function and regulatory mechanisms

To ensure the comparability and reproducibility of results, the expression levels of target genes must be standardized using a set of stably expressed reference genes. However, numerous studies have demonstrated that the expression stability of commonly used reference genes, such as *ACTIN*, *RPL*, *Tubulin*, *GAPDH*, *RPS*, and *EF1A*, exhibits significant variability under different biological or abiotic stress conditions [14,15]. This context-dependent instability underscores the impracticality of using a single “universal” reference gene for accurate normalization across different experimental scenarios [16,17,18,19,20]. The selection of optimal reference genes must therefore be empirically determined and is highly species- and condition-specific. For instance, in studies on *Ericerus pela*, *βTub-2*, *SdhA-1*, and *βTub-1* were identified as the most suitable reference genes for different developmental stages, body parts, and temperature treatments, respectively [21]. For *Maruca vitrata*, RT-qPCR assessments revealed that *RP49* and *RPL13* are the best reference genes for studying the expression of target genes at different developmental stages. When *M. vitrata* is fed with different diets, *RP49* and *RPL24* serve as ideal reference genes, whereas for cross-gender analysis and comparison, *GAPDH* and *RPL24* are the preferred reference genes [22]. For *Chrysoperla nipponensis*, it was found that *Arp3* and *RpS5* are the most stable reference genes across different developmental stages, while *Arp3* and *Tub1* are the most stable reference genes among different adult tissues [23]. These variations highlight that even commonly used reference genes can exhibit inconsistent expression stability within the same species, depending on specific biological contexts such as developmental stage, tissue type, gender, or even under identical experimental conditions [24]. Improper selection of reference genes can lead to biased data on target gene expression or even erroneous conclusions [25]. Therefore, rigorous validation is crucial for specific research objects and experimental conditions [16].

Given the relative scarcity of research on reference genes in the non-biting midge *Propsilocerus akamusi*, which has hindered in-depth analysis of its molecular mechanisms in response to environmental stresses, this study aims to systematically screen and validate the optimal combination of reference genes for this species under different developmental stages, tissue types, as well as temperature, pesticide, and heavy metal stress conditions. This will provide a standardized basis for accurately elucidating its molecular regulatory mechanisms under adverse environmental stresses in future research.

## 2. Methods

### 2.1. Sample Acquisition and Cultivation

The larvae samples of *P. akamusi* were purchased from the Tianjin Flower, Bird, Fish, and Insect Market (the Caozhuang Flower Market in Tianjin is located 100 m south of the intersection of Feixia Road and Fujin Road, Zhongbei Town, Xiqing District). After acquisition, professional morphological identification techniques were employed to screen the larvae one by one, and finally, fourth-instar larvae were selected as the experimental subjects. The fourth-instar larvae were first thoroughly rinsed three times with distilled water to remove impurities and were then transferred to a plastic breeding box containing 1.5 L of distilled water for cultivation. The breeding conditions were as follows: the ventilation rate was precisely controlled at 3 cm, the photoperiod was set to 16–18 h to simulate natural lighting conditions, and the temperature was strictly maintained within the range of 20 ± 1 °C to ensure the stability of the larvae’s growth environment.

Under laboratory conditions, *P. akamusi* larvae can naturally pupate between September and November each year. To facilitate subsequent research, the pupae were promptly subjected to cryopreservation in liquid nitrogen to maintain their biological activity.

For the collection of adult *P. akamusi*, we opted to conduct the capture on the exterior walls of the library (located at 117.118778 degrees east longitude and 39.061474 degrees north latitude) near Qiushui Lake at Tianjin Normal University during the period from October to November each year. The captured adults were randomly transferred to specialized mesh cages in the laboratory for breeding. To simulate natural egg-laying conditions, the mesh cages were placed in containers containing approximately 3 cm deep distilled water, providing suitable conditions for the adults to lay eggs. After the adults laid eggs, the eggs were also preserved in liquid nitrogen for subsequent experimental use.

### 2.2. Sample Processing

Samples of the *P. akamusi* at various developmental stages (egg, larva, pupa, adult) were collected from laboratory cultures. Five biological replicates were established per stage, with each replicate comprising one individual. Field-collected adults were dissected under a stereomicroscope to separate body parts (head, thorax, and abdomen). Five replicates were prepared per body part, each containing five samples. In the temperature stress experiment, 50 fourth-instar larvae were placed in 1.5 L of distilled water and treated for 12 h at temperatures of 4 °C, 15 °C, 25 °C, or 37 °C. Meanwhile, 50 adults were exposed to ambient air and treated for 12 h at temperatures of 4 °C, 25 °C, or 37 °C. Mortality was recorded, and surviving specimens were collected, with five replicates per temperature treatment for both larvae and adults, each replicate containing one individual. Heavy metal stress experiments exposed 50 fourth-instar larvae to 1 L of nickel(II) chloride hexahydrate solution at concentrations of 0, 5, 15, or 20 mmol/L for 120 h. Insecticide stress experiments similarly exposed larvae to deltamethrin solutions at 0, 10, 20, or 50 μg/L. For both chemical treatments, larval mortality was recorded after exposure and survivors collected, with five replicates per concentration, each containing one larva. All collected samples were immediately flash-frozen in liquid nitrogen. RNA was extracted, reverse-transcribed into cDNA, and stored for RT-qPCR analysis.

### 2.3. RNA Extraction (Vazyme Kit)

We use the FastPure Cell/Tissue Total RNA lsolation Kit V2 (*Vazyme*) to extract RNA. Here are the steps: transfer liquid nitrogen-ground chironomid powder into Buffer RL (500 μL/10–20 mg), and vortex until homogeneous. Centrifuge lysate through FastPure gDNA-Filter Column III at 13,400× *g* for 30 s; discard column. Add absolute ethanol (0.5× filtrate vol.) to filtrate. Load onto FastPure RNA Column III, centrifuge at 13,400× *g* for 30 s, and discard flow-through. Wash column with 700 μL Buffer RW1 (13,400× *g*, 30 s), then twice with Buffer RW2 (700 μL, 500 μL; 13,400× *g* for 30 s and 2 min), discarding flow-through each time. Briefly centrifuge if needed to dry the column. Transfer to a new RNase-free tube, elute RNA with 50–200 μL RNase-free ddH_2_O (RT, 1 min; 13,400× *g*, 1 min). Use RNA immediately or store at −85 to −65 °C.

### 2.4. cDNA Synthesis

For the standard protocol, denature 2 µg of total RNA in 8 µL of RNase-free ddH_2_O at 65 °C for 5 min, then chill on ice for 2 min. Add 2 µL of 5× gDNA wiper mix and incubate at 42 °C for 2 min. Set up the cDNA synthesis reaction by adding 2 µL of 10× RT Mix, 2 µL of HiScript III enzyme mix, 1 µL of Oligo (dT)20VN, and 5 µL of RNase-free ddH_2_O to the mixture; incubate at 25 °C for 5 min and 37 °C for 45 min, then inactivate at 85 °C for 5 s. Use the cDNA immediately or store it at −20 °C for up to 6 months; for long-term storage, aliquot and store at −70 °C, avoiding freeze–thaw cycles. For the qPCR-specific protocol, mix 2 µg of total RNA, 2 µL of 5× gDNA wiper mix, and RNase-free ddH_2_O to a total volume of 10 µL, incubate at 42 °C for 2 min, then directly add 2 µL of 10× RT Mix, 2 µL of HiScript III enzyme mix, 1 µL of Oligo (dT)20VN, 1 µL of random hexamers, and 4 µL of RNase-free ddH_2_O. Perform cDNA synthesis at 37 °C for 15 min followed by enzyme inactivation at 85 °C for 5 s, and use the cDNA immediately for qPCR or store it as described above.

### 2.5. Primer Design and Validation

The full-length target gene sequence was used to design forward and reverse primers using Primer 5 software (the primer sequences are shown in Table S1). Primer specificity was confirmed via single-peak melting curves, and amplification efficiency was validated through standard curve analysis. Primers were synthesized by Sangon Biotech with PAGE purification.

### 2.6. RT-qPCR

cDNA templates were reverse-transcribed using Vazyme kits. RT-qPCR reactions (20 μL total volume) contained the following: 10.0 μL 2× ChamQ Universal SYBR RT-qPCR Master Mix, 0.4 μL of each primer (10 μM), 2 μL cDNA, and 7.2 μL RNase-Free ddH_2_O. After brief centrifugation (4 °C, 800 rpm, 5 min), reactions were run with the following program: 95 °C for 30 s (initial denaturation); 40 cycles of 95 °C for 10 s and 60 °C for 30 s; melting curve analysis (60 °C to 95 °C).

### 2.7. COI Gene PCR and Sequencing

COI amplification (25 μL reaction) used TaKaRa Taq™ HS Perfect Mix (12.5 μL), 1.0 μL each primer (5 μM), 8.5 μL of RNase-free ddH_2_O, and 2.0 μL of cDNA. PCR conditions: First, 95 °C for 5 min; and then 35 cycles of 94 °C for 45 s, 58 °C for 45 s, 72 °C for 1 min; final extension at 72 °C for 5 min. PCR products of expected size were verified by agarose gel electrophoresis and sent for sequencing. Sequence alignment with reference was performed using SnapGene 6.0.2.

### 2.8. Date Analysis

The raw Ct values obtained from quantitative PCR (qPCR) were analyzed to assess the stability of reference genes using three different analytical methods: geNorm, BestKeeper, and Normfinder [13,26,27]. Finally, RefFinder was employed to evaluate the comprehensive ranking of reference genes under each condition [28].

### 2.9. Selection of Candidate Reference Genes and Primer Design

Based on studies of reference genes in other insect orders, we selected 15 candidate reference genes in *P. akamusi*: *EF1*, *ACTIN*, *α-TUBLIN*, *RPL32*, *RPL13*, *RPL8*, *RPS17*, *GAPDH*, *RPL27*, *RPS20*, *β-TUBLIN*, *EIF-2α*, *RPS3*, and *RPS11* [14,15]. Given that our research group has already obtained the whole-genome sequence of *P. akamusi*, we retrieved the gene sequences and amino acid sequences of these candidate reference genes. By aligning the amino acid sequences of 15 corresponding reference genes from *P. akamusi* with those of *Drosophila melanogaster* using DNAMAN 8 software, we identified reference genes in *P. akamusi* that exhibited high sequence identity with their *D. melanogaster* counterparts. The gene similarity ranged from 88% to 99%. Subsequently, we amplified these gene fragments using the PCR method and determined their precise sequences through sequencing.

After determining the sequences of the candidate reference genes, we amplified and validated the accuracy of their sequences. The primers used for validation, along with their sequence lengths, are listed in Table 1. We designed primers for RT-qPCR analysis of the 15 candidate reference genes. The specificity of these primers was verified by observing the melting curves, and their amplification efficiency was assessed using the standard curve method. All primers exhibited a single peak in their melting curves, with slopes ranging from −3.623 to −2.800, R^2^ values between 0.986 and 0.999, and amplification efficiencies from 88.81% to 127.58%. Detailed information is provided in Table 1. Detailed primer information is provided in Table S1.

## 3. Results

### 3.1. The Expression Levels of 15 Candidate Reference Genes

**Figure 1:**
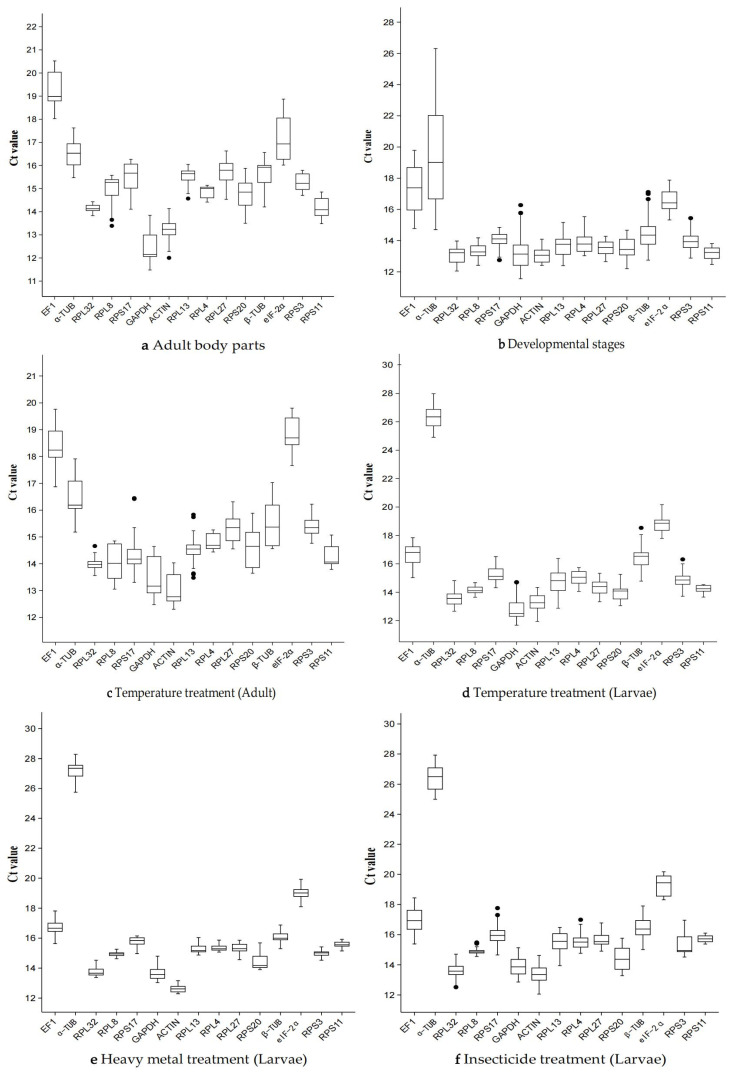
Expression stability of fifteen candidate reference genes in *P. akamusi* under six experimental conditions, presented as raw quantification cycle (Ct values). The panels depict: (**a**) Adult body parts, (**b**) developmental stages, (**c**) temperature treatment of adults, (**d**) temperature treatment of larvae, (**e**) nickel chloride (NiCl_2_) treatment of larvae, and (**f**) cypermethrin (insecticide) treatment of larvae. The *x*-axis lists the fifteen reference genes evaluated (abbreviated gene symbols), whereas the *y*-axis indicates the corresponding Ct value.

The expression levels (Ct values) of 15 candidate reference genes were detected by RT-qPCR across three adult body parts (head, thorax, and abdomen), four developmental stages (egg, larva, pupa, and adult), various temperature treatments (larvae: 0 °C, 15 °C, 25 °C, and 37 °C; adults: 4 °C, 25 °C, and 37 °C), as well as treatments with the heavy metal nickel chloride and the insecticide deltamethrin (For details of Ct values, please refer to Tables S2–S7). All 15 candidate reference genes were expressed under the different treatment conditions mentioned above. (Figure 1). Among the three different adult body parts, *RPL32* exhibited the least variation in expression, while *EF1* and *EIF-2α* showed the greatest variation (Figure 1a). Across different developmental stages, *α-TUB* displayed the most significant variation in expression, whereas *RPL8* and *RPS11* showed the least variation (Figure 1b). In experiments involving different temperature treatments of adults, *RPL32* demonstrated the smallest variation in expression, while in experiments with larvae subjected to different temperature treatments, *RPL8* and *RPS11* showed the least variation (Figure 1c,d).

Under heavy metal exposure, larval samples exhibited significant expression variability in *α-TUB* and *RPS20*, while other tested genes demonstrated minimal expression fluctuations (Figure 1e). In contrast, deltamethrin-treated larvae showed the greatest expression variation in *EF1*, *α-TUB*, and *EIF-2α*, whereas RPL8 and RPS11 displayed the most stable expression profiles (Figure 1f).

### 3.2. Analysis of the Expression Stability of 15 Reference Genes in Different Body Parts of Adults

The geNorm algorithm, which is specifically designed to analyze reference gene stability, primarily evaluates candidate genes’ expression stability through M-values and V*n*/V*n* + 1. A lower M-value indicates higher stability. Additionally, the geNorm 3.5 software can determine the optimal number of reference genes required using the V-value. If V*n*/V*n* + 1 is less than 0.15, then *n* reference genes should be introduced for optimal combination, which helps reduce systematic bias.

**Figure 2:**
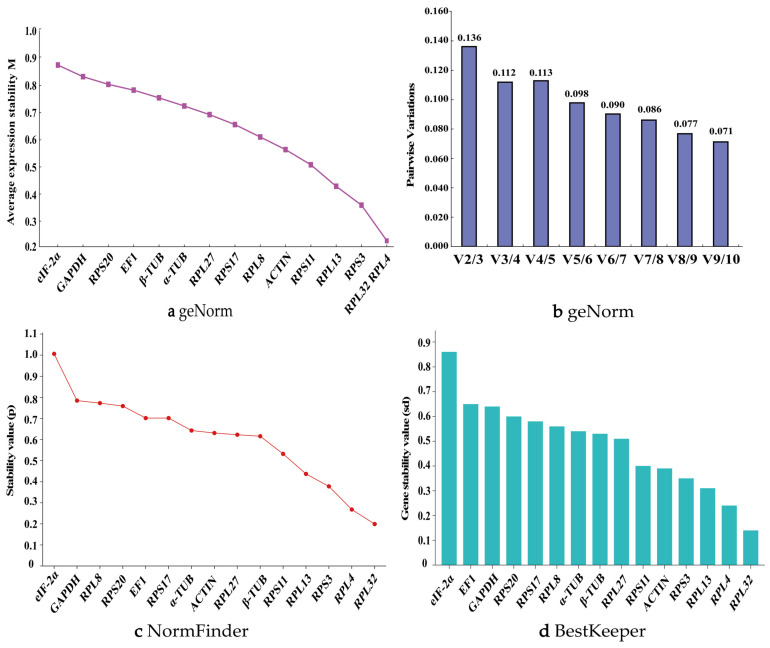
Expression stability of 15 candidate reference genes in *P. akamusi* adults across different body parts. Head, thorax, abdomen dissected from *P. akamusi* adults. The expression stability rankings were determined by (**a**), geNorm, (**b**) geNorm, (**c**) NormFinder, and (**d**) BestKeeper. The geNorm algorithm evaluates the candidate reference genes based on their expression stability values (M-values) in panel a and pairwise variations (V*n*/V*n* + 1) in panel (**b**).

In the experiments involving the head, thorax, and abdomen of adults, genes with M-values less than 0.5 include *RPL32*, *RPS3*, and *RPL13*, with *RPL32* being one of the two most stable genes (Figure 2a). The pairwise variation value V2/3 is 0.136, which is less than 0.15, indicating that two reference genes, including *RPL32*, are suitable for analyzing the expression levels of other genes in different adult body parts (Figure 2b).

The NormFinder algorithm screens for suitable reference genes by measuring the stability value of each gene. A lower stability value indicates better stability, and the gene with the smallest stability value is ultimately selected as the reference gene. In different body parts of adults, *RPL32* has the smallest *p*-value, indicating the most stable expression. Only one gene, *EIF-2α*, has a *p*-value greater than 1.0, making it the least stable gene (Figure 2c).

The BestKeeper algorithm compares the Ct values of each gene to generate paired correlation coefficients (r), coefficients of variation (CV), and standard deviations (SD). The genes are then ranked based on the magnitude of the standard deviation and coefficient of variation, with smaller values indicating better stability. The BestKeeper analysis results show that the stability ranking of the candidate reference genes is as follows: *RPL32*, *RPL13*, *RPS3*, *ACTIN*, *RPS11*, *RPL27*, *β-TUB*, *α-TUB*, *RPL8*, *RPS17*, *RPS20*, *GAPDH*, *EF1*, and *EIF-2α* (Figure 2d).

**Figure 3:**
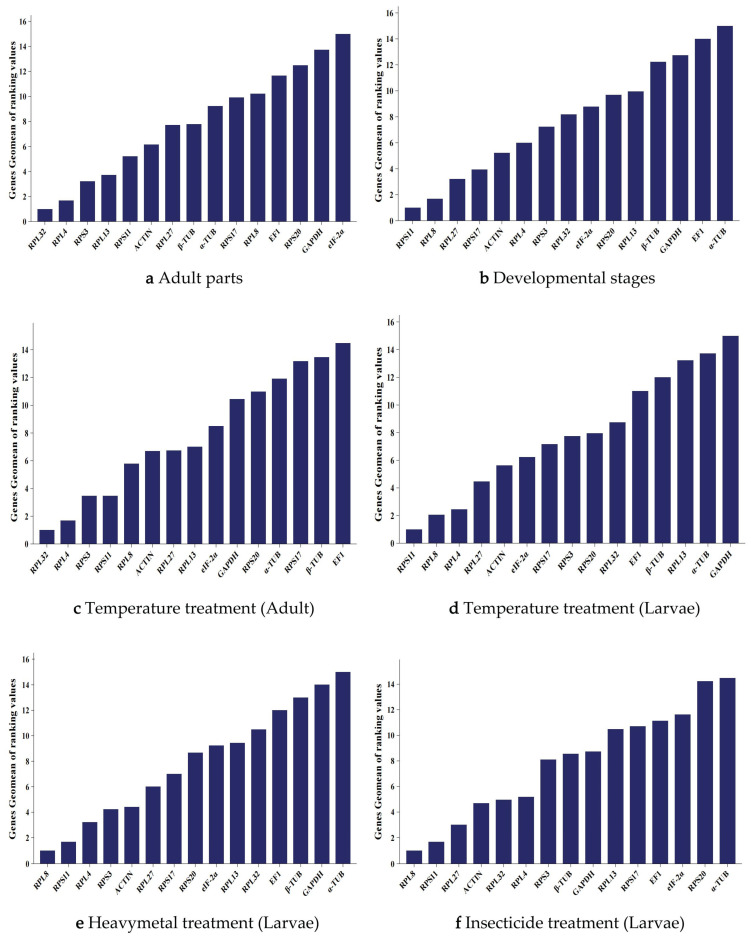
Ranking of the expression stability of 15 candidate reference genes in *P. akamusi* under different conditions. Comprehensive ranking of reference gene expression stability under different conditions by RefFinder. (**a**) Adult tissue. (**b**) Developmental stages. (**c**) Temperature treatment (adults). (**d**) Temperature treatment (larvae). (**e**) NiCl_2_ treatment (larvae). (**f**) Cypermethrin treatment (larvae).

The online tool RefFinder integrates the above algorithms to rank the stability of 15 candidate reference genes. The expression stability ranking is as follows: *RPL32* >> *RPS3* > *RPL13* > *RPS11* > *ACTIN* > *RPL27* > *β-TUB* > *α-TUB* > *RPS17* > *RPL8* > *EF1* > *RPS20* > *GAPDH* > *EIF-2α* (Figure 3a). Consequently, *RPL32* is identified as the most stably expressed reference gene for studies involving different body parts of adults. Supporting data are available in Table S2.

### 3.3. Analysis of the Expression Stability of 15 Reference Genes Across Different Developmental Stages

**Figure 4:**
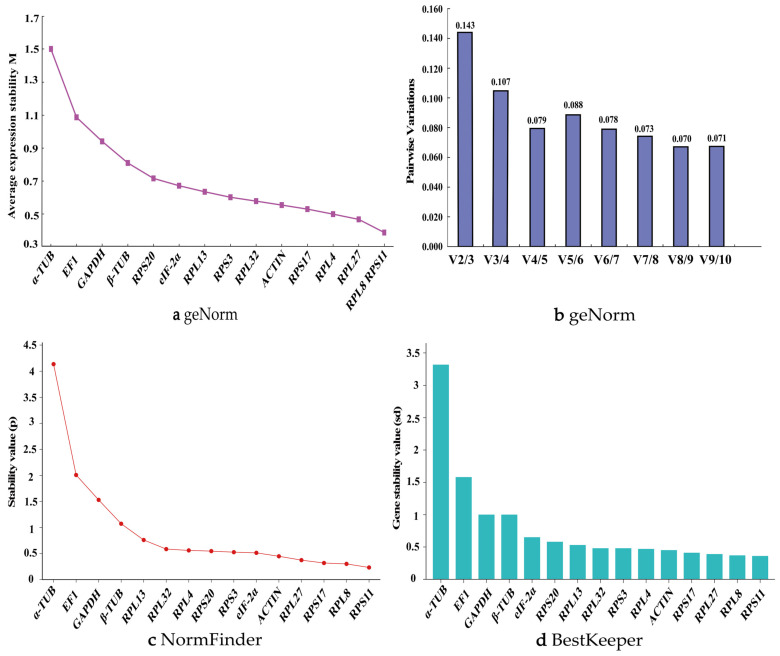
Expression stability of 15 candidate reference genes in *P. akamusi* across different developmental stages. (**a**) geNorm, (**b**) geNorm, (**c**) NormFinder, and (**d**) BestKeeper.

During different developmental stages of *P. akamusi*, the geNorm algorithm revealed that the stability ranking of the 15 candidate reference genes was as follows: *RPL8* > *RPS11* > *RPL27* > *RPL4*> *RPS17* > *ACTIN* > *RPL32* > *RPS3* > *RPL13* > *eIF2α* > *RPS20* > *β-TUB* > *GAPDH* > *EF1* > α-TUB (Figure 4a). Only *EF1* and *α-TUB* had M-values greater than 1, indicating the least stability. *RPL8*, *RPS11*, *RPL27*, and *RPL4* all had M-values less than 0.5, demonstrating the highest stability. The V2/3 value was less than 0.15, suggesting that the combination of two reference genes provides the highest stability (Figure 4b). Therefore, *RPL8* and *RPS11* can be considered the most suitable reference genes during different developmental stages of *P. akamusi*.

The NormFinder algorithm showed that the expression stability ranking of the 15 candidate reference genes across different developmental stages, from highest to lowest, was as follows: *RPS11*, *RPL8*, *RPS17*, *RPL27*, *EIF-2α*, *ACTIN*, *RPS3*, *RPL4*, *RPL32*, *RPS20*, *RPL13*, *β-TUB*, *GAPDH*, *EF1*, and *α-TUB* (Figure 4c).

The BestKeeper analysis results revealed that the stability ranking of the candidate reference genes was as follows: *RPS11*, *RPL8*, *RPS27*, *RPL17*, *ACTIN*, *RPL4*, *RPS3*, *RPL32*, *RPL13*, *RPS20*, *EIF-2α*, *GAPDH*, *β-TUB*, *EF1*, and *α-TUB*. Among these genes, except for *EF1* and *α-TUB*, the Cp values of all other genes were less than 1. The Cp values of *RPS11*, *RPL8*, *RPS27*, *RPL17*, *ACTIN*, *RPS3*, and *RPL32* were all less than 0.5 (Figure 4d). *RPS11* and *RPL8* continued to demonstrate good stability.

Finally, RefFinder provided a comprehensive ranking of the 15 reference genes, indicating that the order was as follows: *RPS11* > *RPL8* > *RPL27* > *RPS17* > *ACTIN* > *RPL4* > *RPS3* > *RPL32* > *EIF2α* > *RPS20* > *RPL13* > *β-TUB* > *GAPDH* > *EF1* > *α-TUB* (Figure 3b). Therefore, in different body parts of *P. akamusi*, *RPL8* and *RPS11* are the most stably expressed reference genes. Supporting data are available in Table S3.

### 3.4. Analysis of the Expression Stability of 15 Reference Genes Under Different Temperature Treatments

**Figure 5:**
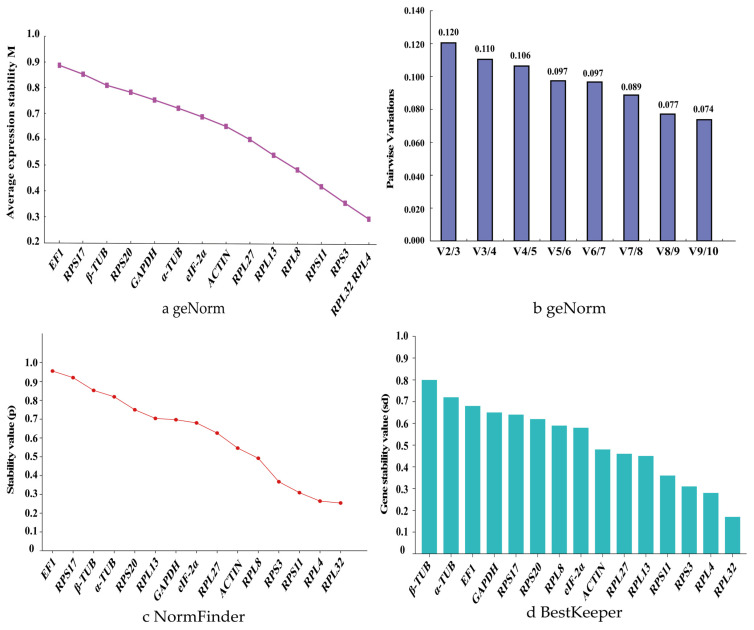
Expression stability of 15 candidate reference genes in *P. akamusi* adults under different temperature conditions. (**a**) geNorm, (**b**) geNorm, (**c**) NormFinder, and (**d**) BestKeeper.

**Figure 6:**
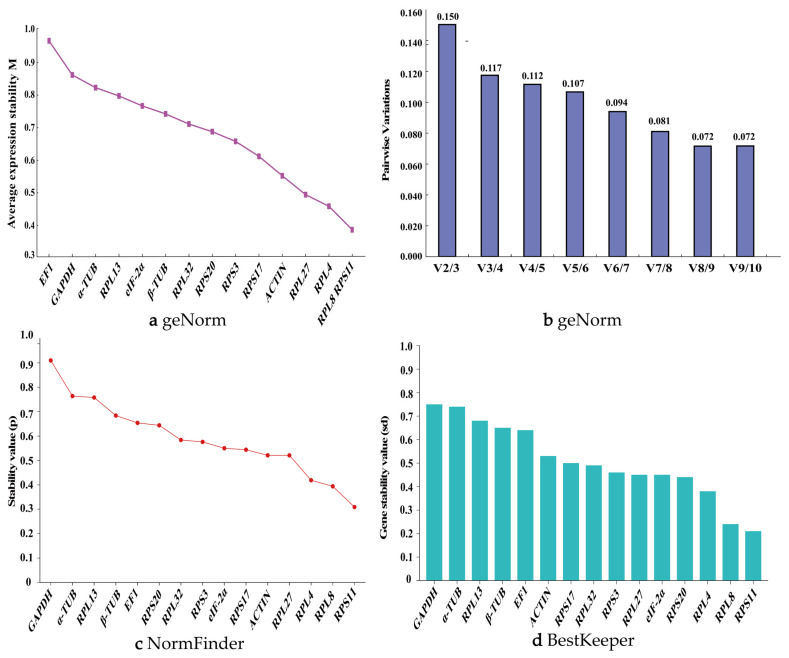
Expression stability of 15 candidate reference genes in *P. akamusi* larvae under different temperature conditions. (**a**) geNorm, (**b**) geNorm, (**c**) NormFinder, and (**d**) BestKeeper.

The geNorm algorithm indicated that, when adult insects were treated with different temperatures, the genes with M-values less than 0.5 included *RPL8*, *RPS11*, *RPS3*, *RPL4*, and *RPL32*. Among these, *RPL32* and *RPL4* were identified as the most stably expressed reference genes (Figure 5a). The V2/3 to V9/10 values were all below 0.15, suggesting that selecting two reference genes is most appropriate for evaluating gene expression levels in adults under different temperature conditions (Figure 5b). When larvae were treated with different temperatures, the genes with M-values less than 0.5 included *RPL27*, *RPL4*, *RPL8*, and *RPS11*, with *RPL8* and *RPS11* being the most stably expressed reference genes (Figure 6a). The V2/3 value was 0.15, indicating that selecting two reference genes is most suitable for assessing gene expression levels in larvae under different temperature conditions (Figure 6b).

The NormFinder algorithm showed that, under different temperature treatments of adults, the stability ranking of the 15 candidate reference genes from highest to lowest was as follows: *RPL32*, *RPL4*, *RPS11*, *RPS3*, *RPL8*, *ACTIN*, *RPL27*, *EIF-2α*, *GAPDH*, *RPL13*, *RPS20*, *α-TUB*, *β-TUB*, *RPS17*, and *EF1*. Among these, *RPL32*, *RPL4*, *RPS11*, *RPS3*, and *RPL8* all had *p*-values less than 0.5 (Figure 5c), indicating high stability. Under different temperature treatments of larvae, the stability ranking of the 15 candidate reference genes from highest to lowest was as follows: *RPS11*, *RPL8*, *RPL4*, *ACTIN*, *RPL27*, *RPS17*, *EIF-2α*, *RPS3*, *RPL32*, *RPS20*, *EF1*, *β-TUB*, *RPL13*, *α-TUB*, and *GAPDH*. Among these, *RPS11* and *RPL8* had *p*-values less than 0.5 (Figure 6c).

The BestKeeper analysis revealed that, under different temperature treatments of adults, *RPL32*, *RPL4*, *RPS11*, and *RPS3* were the most stable candidate reference genes, with Cp values of 0.169, 0.283, 0.310, and 0.357, respectively (Figure 5d). Under different temperature treatments of larvae, *RPS11*, *RPL8*, and *RPL4* were the most stable candidate reference genes, with Cp values of 0.208, 0.242, and 0.378, respectively (Figure 6d).

The comprehensive analysis by RefFinder showed that, under different temperature treatments of adults, the stability ranking of the reference genes was as follows: *RPL32* > *RPL4* > *RPS3* > *RPS11* > *RPL8* > *ACTIN* > *RPL27* > *RPL13* > *EIF-2α* > *GAPDH* > *RPS20* > *α-TUB* > *RPS17* > *β-TUB* > *EF1* (Figure 3c). Under different temperature treatments of larvae, the stability ranking of the reference genes was as follows: *RPS11* > *RPL8* > *RPL27* > *RPS17* > *ACTIN* > *RPL4* > *RPS3* > *RPL32* > *EIF-2α* > *RPS20* > *RPL13* > *β-TUB* > *GAPDH* > *EF1* > *α-TUB* (Figure 3d). Therefore, under different temperature conditions, *RPL32* and *RPL4* were the most stably expressed reference genes in adults of *P. akamusi*, while *RPS11* and *RPL8* were the most stably expressed reference genes in larvae. Supporting data are available in Tables S4 and S5.

### 3.5. Analysis of the Expression Stability of 15 Reference Genes Under Treatments with Heavy Metals and Pesticides

**Figure 7:**
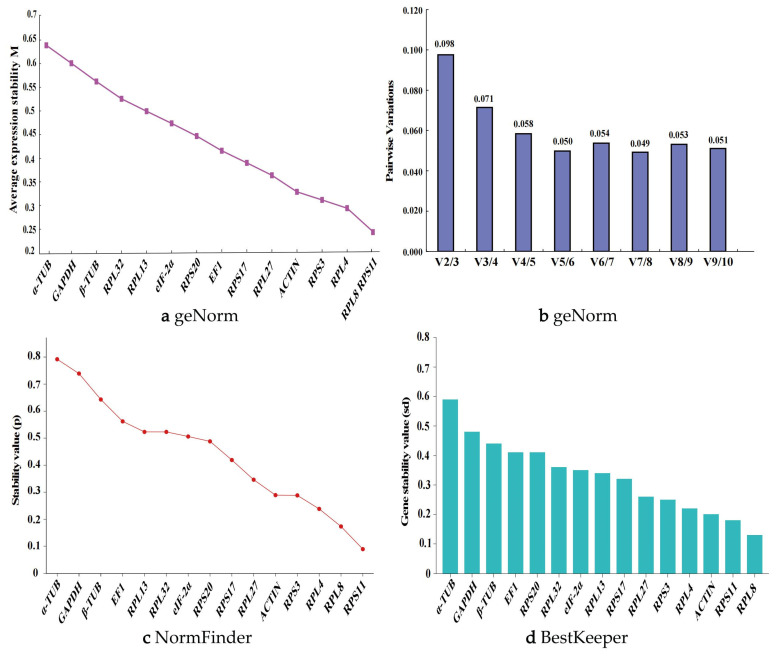
Expression stability of 15 candidate reference genes in *P. akamusi* larvae under NiCl_2_ treatment. (**a**) geNorm, (**b**) geNorm, (**c**) NormFinder, and (**d**) BestKeeper.

**Figure 8:**
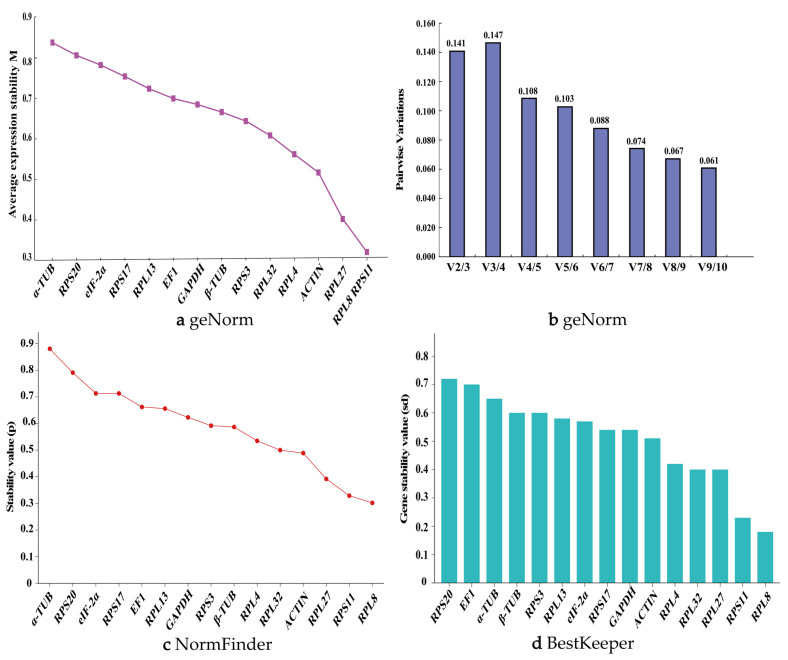
Expression stability of 15 candidate reference genes in *P. akamusi* larvae under Deltamethrin treatment. (**a**) geNorm, (**b**) geNorm, (**c**) NormFinder, and (**d**) BestKeeper.

The geNorm algorithm indicated that, when larvae were treated with nickel chloride, *RPS3*, *RPS11*, and *RPL8* were identified as the most stably expressed reference genes (Figure 7a). The V2/3 to V9/10 values were all below 0.15, suggesting that selecting two reference genes is most appropriate for evaluating gene expression levels in larvae under nickel chloride treatment (Figure 7b). When larvae were treated with the pesticide deltamethrin, the genes with M-values less than 0.5 included *RPL27*, *RPL8*, and *RPS11*, with *RPL8* and *RPS11* being the most stably expressed reference genes (Figure 8a). The V2/3 value was 0.15, indicating that selecting two reference genes is most suitable for assessing gene expression levels in larvae under deltamethrin treatment (Figure 8b). Overall, under treatments with nickel chloride and deltamethrin, *RPS11* and *RPL8* were the most stably expressed reference genes in larvae of *P. akamusi.*

The NormFinder algorithm showed that, under nickel chloride treatment of larvae, the stability ranking of the 15 candidate reference genes from highest to lowest was as follows: *RPL8*, *RPS11*, *RPL4*, *RPS3*, *ACTIN*, *RPL27*, *RPS17*, *RPS20*, *EIF-2α*, *RPL13*, *RPL32*, *EF1*, *β-TUB*, *GAPDH*, and *α-TUB*. Among these, *RPL8*, *RPS11*, *RPL4*, *RPS3*, *ACTIN*, *RPL27*, *RPS17*, and *RPS20* all had *p*-values less than 0.5 (Figure 7c), indicating high stability. Under deltamethrin treatment of larvae, the stability ranking of the 15 candidate reference genes from highest to lowest was as follows: *RPL8*, *RPS11*, *RPL27*, *ACTIN*, *RPL32*, *RPL4*, *β-TUB*, *RPS3*, *GAPDH*, *RPL13*, *EF1*, *EIF-2α*, *RPS17*, *RPS20*, and *α-TUB*. Among these, *RPL8*, *RPS11*, *RPL27*, and *ACTIN* had *p*-values less than 0.5 (Figure 8c).

The BestKeeper analysis revealed that, under nickel chloride treatment of larvae, *RPL8*, *RPS11*, *ACTIN*, *RPL4*, *RPS3*, and *RPL27* were the most stable candidate reference genes, with Cp values of 0.130, 0.178, 0.199, 0.223, 0.245, and 0.262, respectively (Figure 7d). Under deltamethrin treatment of larvae, *RPS11*, *RPL8*, and *RPL27* were the most stable candidate reference genes, with Cp values of 0.178, 0.229, and 0.395, respectively (Figure 8d). Supporting data are available in Tables S6 and S7.

The comprehensive analysis by RefFinder showed that, under nickel chloride treatment of larvae, the stability ranking of the reference genes was as follows: *RPL8* > *RPS11* > *RPL4* > *RPS3* > *ACTIN* > *RPL27* > *RPS17* > *RPS20* > *EIF-2α* > *RPL13* > *RPL32* > *EF1* > *β-TUB* > *GAPDH* > *α-TUB* (Figure 3e). Under deltamethrin treatment of larvae, the stability ranking of the reference genes was as follows: *RPL8* > *RPS11* > *RPL27* > *ACTIN* > *RPL32* > *RPL4* > *RPS3* > *β-TUB* > *GAPDH* > *RPL13* > *RPS17* > *EF1* > *EIF-2α* > *RPS20* > *α-TUB* (Figure 3f). Therefore, under treatments with nickel chloride and deltamethrin, *RPS11* and *RPL8* were the most stably expressed reference genes in larvae of *P. akamusi*. This pair of reference genes is suitable for quantitative analysis of gene expression regulation in larvae under exposure to heavy metals (nickel) and pesticides (deltamethrin), ensuring the reliability of expression data for stress-responsive target genes.

## 4. Discussion

*P. akamusi* is an important aquatic bioindicator species, highly sensitive to water quality changes and widely used in environmental monitoring [4,5]. However, while its ecology and behavior have been relatively well-studied, the molecular response mechanisms remain poorly understood, primarily due to limited genomic resources. The recent completion of its high-quality, chromosome-level genome assembly provides a foundation for functional gene research. Subsequent validation of gene expression and functional studies, however, requires precise gene expression quantification. The reliability of RT-qPCR, a key technique for this purpose, critically depends on stably expressed reference genes [13]. Therefore, identifying and validating optimal reference gene combinations suitable for diverse experimental conditions in *P. akamusi* is essential for in-depth research into its molecular regulatory mechanisms [16].

The 15 candidate reference genes were selected based on their established applicability in insect molecular biology and functional representation. These genes, validated as common reference standards in diverse insect species, encompass fundamental cellular processes such as *GAPDH*, cytoskeletal structure such as *ACTIN* and *α/β-TUBULIN*, and protein synthesis via ribosomal protein genes such as *RPL* and *RPS* [29,30,31,32,33].

No single reference gene is universally applicable under all conditions. While the housekeeping nature of reference genes ensures their expression under most basal conditions, their expression levels are modulated by the functional demands of different cell types and by cellular state changes induced by experimental treatments or environmental stress. Therefore, when selecting reference genes, their robustness must be validated a priori for the specific experimental system used.

In this study, we found that the *RPL32* and *RPL4* genes exhibited the highest expression stability across different body parts and under various temperature treatments in adult *P. akamusi*, thus recommending them as reference genes under these conditions. Meanwhile, the *RPS11* and *RPL8* genes showed the highest stability across different developmental stages, various temperature treatments in larvae, and under nickel chloride and deltamethrin exposures, making them preferable reference genes under these specific conditions. These findings are relatively consistent with results from other insects. For instance, the *RPL32* gene also demonstrated low expression variability across different developmental stages and tissues in *Blattella germanica*, *Thermobia domestica*, and *Eocanthecona furcellata* and was recommended as the optimal reference gene [34,35,36]. The *RPL4* gene exhibited high stability across different developmental stages and genders in *Ophraella communa* [37]. The *RPS11* gene was selected as the most suitable reference gene during different developmental stages in *Kerria lacca*, *Tuta absoluta*, and *Nilaparvata lugens* [38,39,40]. The *RPL8* gene was evaluated as the most appropriate reference gene under different developmental stages, temperature treatments, and odor stimulus conditions in *Cimex hemipterus* [41]. However, *EIF-2α*, the α-subunit of eukaryotic translation initiation factor 2 (eIF-2), orchestrates global protein synthesis by controlling ternary complex formation and is phosphorylated under cellular stress to suppress cap-dependent translation [42,43]. Owing to its central role in the integrated stress response, its transcript abundance is dynamically modulated by developmental stage, tissue-specific metabolic demands, and environmental stressors such as temperature extremes, heavy metals, and pesticide exposure, resulting in the lowest expression stability observed under our experimental conditions.

The present results indicate that ribosomal protein genes, *RPL32*, *RPL4*, *RPS11*, and *RPL8*, exhibit broad applicability across multiple insect species. Under diverse stress conditions, particularly temperature fluctuations and tissue differentiation, these genes display highly conserved expression stability, thereby safeguarding the insects’ adaptive capacity to complex environments [44,45,46]. To accurately investigate the transcriptional responses of these genes under subsequent stress regimes, robust normalization can be achieved not only through rigorous RT-qPCR validation but also by employing the reference-gene combinations *RPL8*, *RPL4*, and *RPL32* or *RPS17* and *RPL32*, which provide reliable internal standards for future experimental analyses.

This study takes *P. akamusi* as the research subject and systematically screens and validates suitable reference gene combinations for this species under various developmental stages body parts, as well as temperature, pesticide, and heavy metal stress conditions. It provides a standardized foundation for future in-depth research utilizing RT-qPCR technology to explore the molecular regulatory mechanisms of *P. akamusi* under adverse environmental conditions. Given the significance of *P. akamusi* as an aquatic bioindicator species, gene expression studies on this organism are crucial for understanding the impacts of environmental pollution on ecosystems. Through precise quantification of gene expression, we can gain a deeper insight into the response mechanisms of *P. akamusi* to environmental changes, thereby offering scientific evidence for environmental protection and water quality monitoring.

## 5. Conclusions

Although this study has made significant progress in screening and validating stable reference genes for *P. akamusi*, several limitations still exist. Firstly, the experimental conditions were relatively constrained, primarily focusing on laboratory-simulated environmental settings, which may not fully reflect the complex and variable conditions of natural environments. Additionally, the optimal combination of reference genes may vary under different experimental conditions, and this study did not comprehensively cover all possible experimental scenarios. The number of repeated experiments set in this study will also have an impact on the research results, and the analysis of the experimental results will also cause differences and randomness. Therefore, adjustments and validations may be necessary based on actual conditions in specific applications.

Future research can be expanded across multiple dimensions. Firstly, it is essential to broaden the scope of experimental conditions by meticulously simulating complex natural environmental variables, such as the interactive effects of multiple pollutants and dynamic fluctuations in abiotic factors. This approach will enable comprehensive evaluation of reference gene stability and applicability across diverse ecological contexts, thereby enhancing the ecological relevance of research findings. Secondly, leveraging advanced gene editing technologies such as CRISPR/Cas9 for precise functional validation of identified core genes should be conducted. Such validation will facilitate in-depth analysis of their specific action pathways and molecular mechanisms underlying *P. akamusi*’s response to environmental stress, significantly advancing our molecular-level understanding of its resilience to adversity. Ultimately, integrating multi-omics technologies—including genomics, transcriptomics, proteomics, and metabolomics—will enable construction of a comprehensive atlas delineating the molecular regulatory mechanisms underlying *P. akamusi*’s adaptation to adverse conditions. This integrated approach will deepen our comprehension of organismal adaptation and defense mechanisms against environmental stressors, providing robust scientific support for sustainable aquatic ecosystem protection and management.

## Figures and Tables

**Table 1:** Primers for qRT-PCR detection of 15 candidate reference genes.

Gene Name	Primer Sequences (5′-3′)	Length (bp)	Slope	R^2^	Efficiency
*EF1*	F-AACCACCATACTCTGAAACTCG R-AATCAAGCATTTGCCATCAG	212	−3.310	0.998	100.05%
*ACTIN*	F-CCGTCTTCCCATCCATCGT R-ATGTTCCTCTGGGGCGACAC	218	−3.310	0.995	100.05%
*α-TUB*	F-CATTTGGTGGTGGAACAGG R-GCTATGTGTGGTCAGAACGG	161	−2.800	0.993	127.58%
*RPL32*	F-AGGCATTCATTCGCCATCA R-CATCAGAACCTCCAGTTCACG	215	−3.587	0.997	90.01%
*RPL13*	F-TCCAAATGCCCACTTCCA R-GCCTTCAATTCAGCCAATGA	222	−3.548	0.998	91.36%
*RPL8*	F-ACTTCCGTGACCCATACCG R-CCTGATGTTCTTGCCAACTTAC	208	−3.440	0.989	95.30%
*RPS17*	F-CACTCGCTTGACATTGGATT R-ACCACGAACTTGGGAGTGAC	142	−3.520	0.999	91.29%
*GAPDH*	F-ACTGTTCATGCCACCACAGC R-TAACTTTGCCGACAGCCTTG	133	−3.376	0.998	97.79%
*RPL4*	F-TGGCGTAGATGGCACAGAC R-TCGGAAACAACCAATGGAAG	146	−3.550	0.999	91.29%
*RPL27*	F-TGACGGCACATCGGATAAAC R-AACGCTGTAACGTGTTGGCA	172	−3.623	0.997	88.81%
*RPS20*	F-TCGTATTGTCTTGACGGCAT R-GGAAACGATCCCAGGTCTTA	182	−3.282	0.988	101.69%
*β-TUB*	F-GTCTTTGGACAATCGGGAGC R-GATCGGGATATTCCTCACGG	214	−3.490	0.998	93.43%
*EIF-2α*	F-AAATACGAAACGGACGAGCAG R-GCACGAATTTTGACAGCCTG	218	−3.285	0.986	101.56%
*RPS3*	F-TTCATCATGGAATCTGGTGC R-TCCTTGACGCAACAGAACG	168	−3.251	0.997	103.05%
*RPS11*	F-CATCCGCCGTGACTACTTG R-CCAGCGACTTTGTTGACCTT	189	−3.301	0.998	100.88%

## Data Availability

The original contributions presented in this study are included in the article/Supplementary Materials. Further inquiries can be directed to the corresponding author.

## Supplementary Materials

The following supporting information can be downloaded at: https://www.mdpi.com/article/10.3390/biology14091158/s1, Table S1: RT-PCR primers for reference genes; Table S2: CT values measured from different body parts of adult *Propsilocerus akamusi* under various treatment conditions; Table S3: CT values measured from different developmental stages of adult *Propsilocerus akamusi* under various treatment conditions; Table S4: CT values measured from *Propsilocerus akamusi* larvae under different temperature treatment conditions; Table S5: CT values measured from *Propsilocerus akamusi* adult under different temperature treatment conditions; Table S6: CT values measured from *Propsilocerus akamusi* larvae under heavy metal treatment conditions; Table S7: CT values measured from *Propsilocerus akamusi* larvae under deltamethrin treatment conditions.
