# Supplementary material for: Reference Genes for Expression Analyses by qRT-PCR in Propsilocerus akamusi (Diptera: Chironomidae)
Source: Biology (Basel). 2025 Sep 1;14(9):1158. doi: 10.3390/biology14091158 (PMC12467372; doi:10.3390/biology14091158)
Supplement: Supplementary file 1 [file biology-14-01158-s001.zip › Table S1.pdf]

Table S1. RT-PCR primers for reference genes.

Gene name	Primer sequences（5′-3′）	length（bp）
EF1	Forward : AACTGAACCACCATACTCTGA<br>Reverse : CACGACGCAATTCCTTAA	498
ACTIN	Forward : GCTCCGGTATGTGCAAGG<br>Reverse : AAGTGTAGCCACGTTCTGTC	561
α-TUBLIN	Forward : TGTGATGGATTTCAGGGTT<br>Reverse : GCCAATGGAGCATAGGAA	443
RPL32	Forward : TTCAAACCCAGAATCGTCA<br>Reverse : GCATCATCAGAACCTCCAGT	250
RPL13	Forward : GATGATTCCAAATGCCCACT<br>Reverse : TCCTCGGTAGCTTCACCCT	408
RPL8	Forward : ACTTCCGTGACCCATACCG<br>Reverse : CTTGTCGTCTCCCTTTCCA	578
RPS17	Forward : TACTACACTCGCTTGACATTG<br>Reverse : ATCCGGTGATATTGCTGA	292
GAPDH	Forward : GGCTCCATTGGCTAAGGT<br>Reverse : GACTTGTTCTTCGGTGTATTCTA	373
RPL4	Forward : CGCACCAACCAAACCATG<br>Reverse : CGCTTAATTTCTTCAGCCTTC	594
RPL27	Forward : CATTCGATGACGGCACAT<br>Reverse : CGCTTGATTGGGTCTTTC	238
RPS20	Forward : TCGCATTCGTATTGTCTTG<br>Reverse : CACCAGGTTCGATGTTGATT	275
β-TUBLIN	Forward : GACTCGGTTCGTTCAGGT<br>Reverse : ATCGCCGTATGTTGGTGT	453
EIF-2α	Forward : CTGAGCCAGTTGTTGTGATT<br>Reverse : GCCTCAAGTCCAGCGAAT	448
RPS3	Forward : AGGTTGCTACTCGTGGTCTT<br>Reverse : TCGGGATTCTGATAGTTGGT	400
RPS11	Forward : ATCCGCCGTGACTACTTG<br>Reverse : CAGCGACTTTGTTGACCTTC	187
