# Supplementary material for: Reference Genes for Expression Analyses by qRT-PCR in Propsilocerus akamusi (Diptera: Chironomidae)
Source: Biology (Basel). 2025 Sep 1;14(9):1158. doi: 10.3390/biology14091158 (PMC12467372; doi:10.3390/biology14091158)
Supplement: Supplementary file 1 [file biology-14-01158-s001.zip › Table S2.pdf]

Table S2. CT values measured from different body parts of adult *Propsilocerus akamusi* under various treatment conditions

	EF1	*α-TUB*	*RPL32*	*RPL8*	*RPS17*	*GAPDH*	*ACTIN*	*RPL13*	*RPL4*	*RPL27*	*RPS20*	*β-TUB*	*eIF-2α*	*RPS3*	*RPS11*
1	20.196	15.483	14.060	15.474	16.156	12.145	14.136	14.828	15.011	15.813	15.030	15.641	16.018	15.637	14.060
2	18.971	17.254	14.053	15.577	16.064	12.078	13.953	15.709	15.003	15.876	15.019	15.625	16.932	14.703	14.064
3	18.987	16.244	14.432	15.424	15.911	11.825	13.384	15.647	15.024	14.535	14.849	14.206	16.124	15.635	13.481
4	19.971	15.804	14.129	15.327	15.877	11.909	13.555	15.674	15.096	15.793	14.778	14.525	16.157	15.618	13.511
5	18.952	17.290	14.300	15.264	15.620	12.150	13.303	15.353	15.080	15.505	14.810	14.834	16.163	15.790	14.087
6	18.022	16.180	14.251	15.383	15.669	12.259	13.413	15.387	14.669	14.560	14.777	14.899	16.376	14.708	14.040
7	20.039	16.624	14.042	15.385	16.209	12.047	12.986	15.790	14.877	16.371	15.877	15.921	17.278	15.555	13.633
8	20.047	17.624	14.339	15.390	16.053	12.111	13.020	15.731	15.106	16.302	15.750	15.964	17.186	15.226	14.223
9	19.261	16.529	13.830	14.703	15.028	13.169	14.035	15.060	14.652	15.582	13.499	16.002	16.596	14.967	14.838
10	18.535	15.470	13.865	14.834	15.014	12.220	13.202	16.049	14.519	16.484	13.638	16.002	16.573	14.822	14.837
11	18.625	17.623	13.972	14.726	15.223	12.815	13.147	15.870	14.549	15.241	13.790	16.034	18.030	15.688	14.853
12	18.633	16.442	14.065	14.720	15.005	13.847	13.238	15.804	14.417	15.667	13.699	15.913	18.870	15.680	14.742
13	18.955	15.873	14.299	13.858	14.109	11.479	12.972	15.618	15.143	16.628	15.620	16.004	18.565	14.956	14.402
14	20.031	16.597	14.164	13.650	14.211	13.648	12.004	15.558	15.039	14.709	15.298	16.089	18.608	15.135	14.369
15	20.523	16.588	14.160	13.389	16.268	13.797	12.558	14.570	14.491	15.807	15.179	16.564	18.075	15.132	13.593
