# Supplementary material for: Reference Genes for Expression Analyses by qRT-PCR in Propsilocerus akamusi (Diptera: Chironomidae)
Source: Biology (Basel). 2025 Sep 1;14(9):1158. doi: 10.3390/biology14091158 (PMC12467372; doi:10.3390/biology14091158)
Supplement: Supplementary file 1 [file biology-14-01158-s001.zip › Table S3.pdf]

Table S3. CT values measured from different developmental stages of adult *Propsilocerus akamusi* under various treatment conditions

	EF1	*α-TUB*	*RPL32*	*RPL8*	*RPS17*	*GAPDH*	*ACTIN*	*RPL13*	*RPL4*	*RPL27*	*RPS20*	*β-TUB*	*EIF-2α*	*RPS3*	*RPS11*
1	19.754	15.588	13.198	13.805	14.093	13.133	14.093	14.093	13.945	13.767	13.317	14.372	15.805	14.273	13.525
2	16.067	18.585	13.640	14.121	14.638	13.435	14.091	14.091	13.820	13.748	13.429	14.597	16.630	14.597	13.429
3	15.655	25.431	13.077	14.177	14.851	11.992	13.728	13.728	14.442	14.019	14.542	16.996	17.879	14.357	13.813
4	18.321	20.883	13.882	12.770	14.352	14.586	12.573	12.573	13.319	13.787	14.130	13.145	16.119	13.742	12.627
5	19.786	15.763	13.279	13.375	13.852	13.231	12.661	14.007	14.351	13.497	14.357	14.148	16.265	14.249	13.766
6	16.709	16.981	13.395	12.974	13.644	12.765	13.344	13.581	13.622	13.140	12.235	14.046	16.494	13.573	13.455
7	16.564	26.118	13.521	13.259	13.927	11.560	12.642	13.478	14.225	13.249	14.073	17.112	17.729	14.080	13.621
8	18.058	19.742	12.674	12.555	12.756	14.572	12.451	12.967	13.033	12.657	13.816	13.252	16.114	13.063	12.562
9	18.256	15.120	12.054	12.991	13.241	12.764	12.415	15.166	13.317	13.054	12.208	13.398	15.697	14.171	12.782
10	14.818	17.003	13.325	13.840	14.716	13.370	12.841	14.464	15.533	14.207	13.779	14.442	15.867	12.958	13.150
11	14.773	26.001	12.730	13.161	13.863	12.337	12.520	13.012	13.745	13.288	12.566	16.662	17.540	13.820	13.457
12	18.621	19.450	12.398	13.493	13.117	15.779	13.061	12.398	13.198	14.085	12.952	12.751	15.406	14.012	13.263
13	18.925	15.693	13.427	13.254	14.100	13.167	13.068	14.458	14.348	14.278	13.444	13.760	17.324	15.443	13.793
14	16.315	17.215	13.245	13.825	14.180	12.447	13.538	14.133	13.957	13.444	13.128	14.570	17.057	14.346	13.549
15	15.100	25.813	12.087	13.379	14.121	12.261	13.320	13.829	13.514	13.173	12.901	16.417	17.385	13.563	13.223
16	19.696	19.995	12.438	13.623	13.706	15.549	13.998	13.636	13.174	13.102	13.177	14.478	16.114	14.511	12.868
17	18.873	14.706	13.978	13.292	14.131	13.148	12.945	13.822	14.258	14.181	13.633	14.026	16.915	13.859	12.831
18	16.355	17.068	13.875	13.206	14.172	12.877	13.285	13.166	14.068	13.637	13.426	14.339	16.343	13.550	13.045
19	14.836	26.313	13.026	13.054	14.534	12.154	13.195	13.815	13.269	13.876	14.667	15.826	16.963	13.504	12.876
20	18.557	19.873	12.464	12.419	14.553	16.277	12.584	12.947	13.316	13.126	14.326	13.778	15.327	12.883	12.475
