# Supplementary material for: Reference Genes for Expression Analyses by qRT-PCR in Propsilocerus akamusi (Diptera: Chironomidae)
Source: Biology (Basel). 2025 Sep 1;14(9):1158. doi: 10.3390/biology14091158 (PMC12467372; doi:10.3390/biology14091158)
Supplement: Supplementary file 1 [file biology-14-01158-s001.zip › Table S4.pdf]

Table S4. CT values measured from *Propsilocerus akamusi* larvae under different temperature treatment conditions

	EF1	*α-TUB*	*RPL32*	*RPL8*	*RPS17*	*GAPDH*	*ACTIN*	*RPL13*	*RPL4*	*RPL27*	*RPS20*	*β-TUB*	*EIF-2α*	*RPS3*	*RPS11*
1	17.2833	25.157	13.423	13.652	14.318	14.252	13.140	14.645	15.077	15.083	13.893	15.877	17.865	14.668	14.158
2	17.0254	25.616	12.944	14.065	14.921	11.816	14.179	14.026	14.974	15.166	14.053	16.810	18.838	14.886	14.444
3	16.6999	27.283	13.853	14.523	15.647	12.311	13.185	14.046	15.482	14.177	14.121	16.734	18.876	13.774	14.173
4	16.1276	27.212	13.185	14.037	15.087	12.292	12.835	14.258	14.891	14.684	14.902	17.567	19.063	15.588	14.535
5	15.6935	26.264	12.998	13.993	16.294	14.713	12.683	15.312	15.340	14.330	14.230	15.986	18.286	14.838	14.496
6	15.7831	25.829	13.132	13.911	16.481	11.990	13.746	15.827	14.575	13.961	14.184	15.953	17.797	14.580	14.182
7	15.0253	25.990	13.275	14.200	15.058	12.205	12.906	15.838	15.175	14.459	14.151	16.495	19.021	14.658	14.495
8	17.8399	27.968	13.932	14.079	15.111	12.768	13.256	15.493	15.450	14.281	14.226	16.570	18.379	14.442	14.518
9	16.6226	25.746	13.637	14.559	15.130	14.698	12.869	14.148	14.980	14.467	13.059	15.005	19.136	14.840	13.867
10	17.1795	25.564	13.143	13.793	15.307	13.362	14.343	14.946	15.737	15.225	13.076	17.245	20.162	15.138	13.951
11	17.2894	25.893	13.865	14.004	15.528	13.979	13.800	14.747	15.700	15.336	14.226	16.773	18.716	16.316	14.479
12	17.7562	27.973	14.579	14.244	16.513	13.217	13.675	16.379	15.040	14.354	14.528	18.533	19.293	15.719	14.353
13	16.1624	25.215	12.670	14.383	14.372	12.332	11.951	12.881	14.576	13.783	13.414	14.786	17.795	13.718	14.096
14	16.9396	26.435	14.501	14.675	15.007	12.485	14.094	13.597	15.467	14.474	13.480	17.359	18.772	15.083	14.022
15	16.9054	26.720	13.501	14.130	14.818	12.538	13.392	14.814	15.664	14.824	13.567	15.818	19.797	14.931	14.301
16	17.7179	26.678	13.753	14.487	15.934	13.182	13.999	14.988	15.118	14.600	13.547	16.152	18.890	13.753	14.375
17	16.0181	26.416	13.777	14.290	14.748	12.339	13.264	14.805	14.562	13.754	15.265	15.784	18.283	15.152	14.010
18	15.9469	24.907	14.775	13.776	15.130	11.684	12.026	13.841	14.069	13.335	14.313	16.188	18.807	14.933	14.203
19	16.3012	26.759	13.383	13.725	14.749	12.427	12.894	15.544	14.328	13.508	14.058	16.717	19.047	15.140	13.661
20	17.1385	27.464	14.817	14.357	15.653	13.216	13.739	15.305	14.658	13.900	13.479	16.615	19.198	14.516	14.486
