# Supplementary material for: Reference Genes for Expression Analyses by qRT-PCR in Propsilocerus akamusi (Diptera: Chironomidae)
Source: Biology (Basel). 2025 Sep 1;14(9):1158. doi: 10.3390/biology14091158 (PMC12467372; doi:10.3390/biology14091158)
Supplement: Supplementary file 1 [file biology-14-01158-s001.zip › Table S5.pdf]

Table S5. CT values measured from *Propsilocerus akamusi* adult under different temperature treatment conditions

	EF1	*α-TUB*	*RPL32*	*RPL8*	*RPS17*	*GAPDH*	*ACTIN*	*RPL13*	*RPL4*	*RPL27*	*RPS20*	*β-TUB*	*EIF-2α*	*RPS3*	*RPS11*
1	18.324	16.039	14.092	14.015	14.015	12.887	12.580	14.521	14.585	16.046	13.937	14.624	19.806	15.159	13.997
2	18.227	15.954	13.993	14.170	13.959	12.847	12.448	14.690	14.472	16.311	13.780	14.745	18.695	15.199	14.008
3	16.890	17.892	14.134	14.724	13.978	14.491	12.742	14.714	15.103	15.607	15.192	17.029	19.667	16.221	14.223
4	16.871	17.003	14.074	14.541	14.069	12.474	12.782	14.653	15.214	15.617	15.158	17.009	17.869	14.998	15.072
5	18.333	17.885	14.196	14.830	13.304	13.307	12.990	15.830	15.218	14.585	14.649	16.131	18.631	15.768	14.643
6	18.241	17.912	14.662	14.819	13.436	13.354	12.302	15.741	15.262	15.246	14.716	16.247	19.304	15.920	14.636
7	18.232	16.111	13.766	13.469	14.224	14.645	12.545	14.410	15.150	15.348	15.326	14.568	17.665	15.348	14.065
8	18.308	16.090	13.686	13.447	14.282	12.943	12.767	14.418	14.930	14.936	15.862	15.578	18.365	15.336	14.064
9	17.714	16.190	13.836	13.322	14.279	14.337	12.647	14.869	14.440	14.578	13.647	14.773	19.667	15.764	14.325
10	17.724	16.212	13.963	13.376	14.175	12.512	12.645	14.290	14.450	14.556	14.563	14.563	17.886	15.455	14.001
11	19.765	15.184	13.945	14.757	16.419	14.234	13.592	13.606	14.550	15.644	15.886	15.369	18.632	15.476	14.756
12	19.651	15.903	13.559	14.850	16.447	14.307	14.035	13.641	14.591	14.783	14.357	15.746	19.443	15.123	15.016
13	19.569	16.096	13.885	13.590	14.793	13.013	13.766	14.550	14.692	15.897	14.896	14.683	18.793	14.763	13.789
14	19.602	17.100	14.010	13.655	14.869	12.995	13.688	14.563	14.680	15.698	13.763	14.665	18.523	14.884	14.023
15	18.225	17.067	13.978	13.054	14.093	13.167	13.593	13.478	15.001	15.224	13.654	16.785	19.435	15.465	13.814
