# Supplementary material for: Reference Genes for Expression Analyses by qRT-PCR in Propsilocerus akamusi (Diptera: Chironomidae)
Source: Biology (Basel). 2025 Sep 1;14(9):1158. doi: 10.3390/biology14091158 (PMC12467372; doi:10.3390/biology14091158)
Supplement: Supplementary file 1 [file biology-14-01158-s001.zip › Table S6.pdf]

Table S6. CT values measured from *Propsilocerus akamusi* larvae under heavy metal treatment conditions

	EF1	*α-TUB*	*RPL32*	*RPL8*	*RPS17*	*GAPDH*	*ACTIN*	*RPL13*	*RPL4*	*RPL27*	*RPS20*	*β-TUB*	*EIF-2α*	*RPS3*	*RPS11*
1	17.371	28.270	13.374	14.626	14.634	13.225	12.292	15.134	15.465	15.581	14.217	16.270	19.332	14.792	15.403
2	16.786	27.219	13.733	14.808	16.121	13.899	12.666	15.604	15.296	15.785	15.030	15.711	18.870	14.908	15.852
3	16.542	28.081	13.962	14.954	16.057	14.015	12.329	15.537	15.604	15.051	14.818	16.006	19.225	15.047	15.728
4	16.639	26.450	13.494	14.857	15.581	14.100	12.557	15.196	15.158	15.187	14.017	17.298	18.444	14.341	15.144
5	16.998	27.782	13.659	15.077	15.917	13.035	12.554	15.063	15.289	15.278	14.417	15.968	19.280	15.051	15.517
6	16.997	27.340	14.059	15.260	16.146	13.458	12.677	15.503	16.146	15.173	14.779	16.279	19.146	15.713	15.559
7	17.168	26.960	13.632	14.886	15.668	13.364	12.748	14.872	15.088	15.073	13.902	15.932	18.912	14.630	15.162
8	16.667	26.332	15.216	15.197	16.013	15.751	12.404	15.207	15.244	15.271	14.471	17.360	19.011	15.162	15.559
9	17.211	27.369	13.762	14.940	14.663	13.537	12.565	15.284	15.474	15.202	15.682	16.300	19.177	14.895	15.922
10	16.451	25.312	13.924	15.000	15.797	13.218	12.326	15.115	15.232	15.373	15.260	15.097	18.510	15.029	15.555
11	16.231	27.374	13.558	14.997	15.470	13.340	12.880	15.091	15.128	14.563	14.211	16.423	19.331	14.883	15.750
12	16.488	27.061	13.662	14.757	15.166	15.512	12.652	14.950	15.069	14.733	13.998	15.815	18.618	14.780	15.241
13	17.815	28.089	13.406	14.840	15.882	13.133	12.358	14.996	15.494	15.858	14.116	16.045	20.112	15.138	15.793
14	16.945	27.410	13.816	15.135	16.128	13.614	13.161	15.454	16.301	15.031	14.888	15.931	19.009	15.380	15.491
15	16.826	27.477	13.597	14.957	15.924	13.779	12.981	15.080	15.423	15.613	14.134	15.914	19.109	14.998	15.673
16	16.657	26.386	13.499	14.976	15.916	13.945	12.900	15.109	15.394	15.661	14.038	15.284	18.571	15.018	15.670
17	15.301	28.275	13.500	14.842	15.793	13.263	12.419	16.518	15.246	15.498	13.911	15.986	18.835	13.932	15.521
18	15.811	27.467	14.087	15.200	16.142	13.667	12.620	16.950	15.303	15.631	14.047	15.579	19.009	15.267	15.735
19	15.980	27.143	13.553	14.749	15.758	13.390	12.606	15.119	15.215	15.484	14.110	16.089	19.321	15.064	15.489
20	16.448	25.928	15.365	14.847	15.607	13.781	13.066	15.317	15.099	15.320	14.038	17.460	17.372	15.011	15.173
