# Supplementary material for: Reference Genes for Expression Analyses by qRT-PCR in Propsilocerus akamusi (Diptera: Chironomidae)
Source: Biology (Basel). 2025 Sep 1;14(9):1158. doi: 10.3390/biology14091158 (PMC12467372; doi:10.3390/biology14091158)
Supplement: Supplementary file 1 [file biology-14-01158-s001.zip › Table S7.pdf]

Table S7. CT values measured from *Propsilocerus akamusi* larvae under deltamethrin treatment conditions

	EF1	*α-TUB*	*RPL32*	*RPL8*	*RPS17*	*GAPDH*	*ACTIN*	*RPL13*	*RPL4*	*RPL27*	*RPS20*	*β-TUB*	*EIF-2α*	*RPS3*	*RPS11*
1	18.106	26.444	14.704	15.446	16.428	15.085	14.513	16.125	15.874	15.944	15.179	17.283	18.313	16.916	16.100
2	16.283	26.344	14.206	15.114	15.936	14.364	12.898	14.820	15.090	15.438	15.531	16.338	20.169	14.848	15.850
3	18.439	26.593	13.394	14.904	15.378	14.356	13.714	16.439	15.748	15.905	15.073	17.170	19.974	16.957	15.921
4	17.338	25.674	13.883	14.781	15.717	14.005	13.296	15.597	15.309	15.301	14.881	16.407	19.320	15.949	15.394
5	17.591	24.995	13.603	14.901	15.463	15.131	14.091	15.709	16.182	16.027	14.667	17.901	18.368	15.824	16.038
6	17.391	27.182	13.501	14.753	17.300	13.339	13.415	14.077	16.462	16.169	13.409	16.593	19.955	14.913	16.039
7	16.865	27.077	13.561	14.774	15.762	13.196	13.253	15.546	16.111	15.572	15.765	16.911	19.927	14.847	15.543
8	17.694	26.362	13.968	15.081	16.041	13.512	13.939	15.863	15.632	15.396	15.049	16.892	19.878	15.979	15.408
9	17.966	26.627	13.359	14.847	15.760	14.793	14.115	15.085	16.989	16.775	13.279	16.221	18.796	15.473	16.017
10	15.860	26.274	12.517	14.866	15.392	13.880	13.741	15.363	15.592	15.358	15.665	16.568	19.812	14.600	16.073
11	16.363	27.926	13.054	14.785	16.664	13.778	13.043	16.043	15.045	15.768	13.352	15.657	19.339	14.933	15.375
12	16.703	26.555	12.746	14.845	14.654	14.987	13.098	15.571	15.161	14.904	13.725	15.205	18.590	14.516	15.426
13	16.989	25.656	12.811	15.479	16.155	13.380	14.621	16.302	15.425	15.505	14.088	15.748	18.400	15.234	15.671
14	15.379	25.510	13.329	14.712	15.106	12.859	12.058	13.944	15.658	14.272	13.397	15.010	18.453	14.884	15.767
15	16.460	26.731	14.491	15.407	15.958	13.146	12.663	16.210	15.178	15.086	13.639	16.130	19.684	14.750	15.883
16	17.352	27.596	13.954	14.904	17.760	13.846	13.640	16.482	15.379	15.505	14.131	15.915	19.207	14.906	15.504
17	18.318	25.117	13.595	14.824	16.221	13.641	12.916	15.267	15.727	15.966	14.578	17.025	18.454	15.674	15.995
18	16.222	25.439	13.419	14.852	16.749	13.398	12.845	14.704	14.762	16.220	15.282	16.076	19.624	16.071	16.053
19	16.868	27.386	13.665	14.891	16.164	14.188	13.001	15.002	15.005	15.637	14.156	16.017	19.540	14.884	15.646
20	16.334	27.128	13.657	14.829	15.656	13.942	13.403	15.160	15.178	15.378	14.161	17.540	19.975	14.958	15.654
